# Supplementary material for: Towards unified AI-driven fracture mechanics: the extended deep energy method (XDEM)
Source: Nat Commun. 2026 Aug 15;17:8492. doi: 10.1038/s41467-026-76748-1 (PMC13484471; doi:10.1038/s41467-026-76748-1)
Supplement: Supplementary file 3 [file 41467_2026_76748_MOESM3_ESM.zip › pic/Method/Extended_function.pdf]

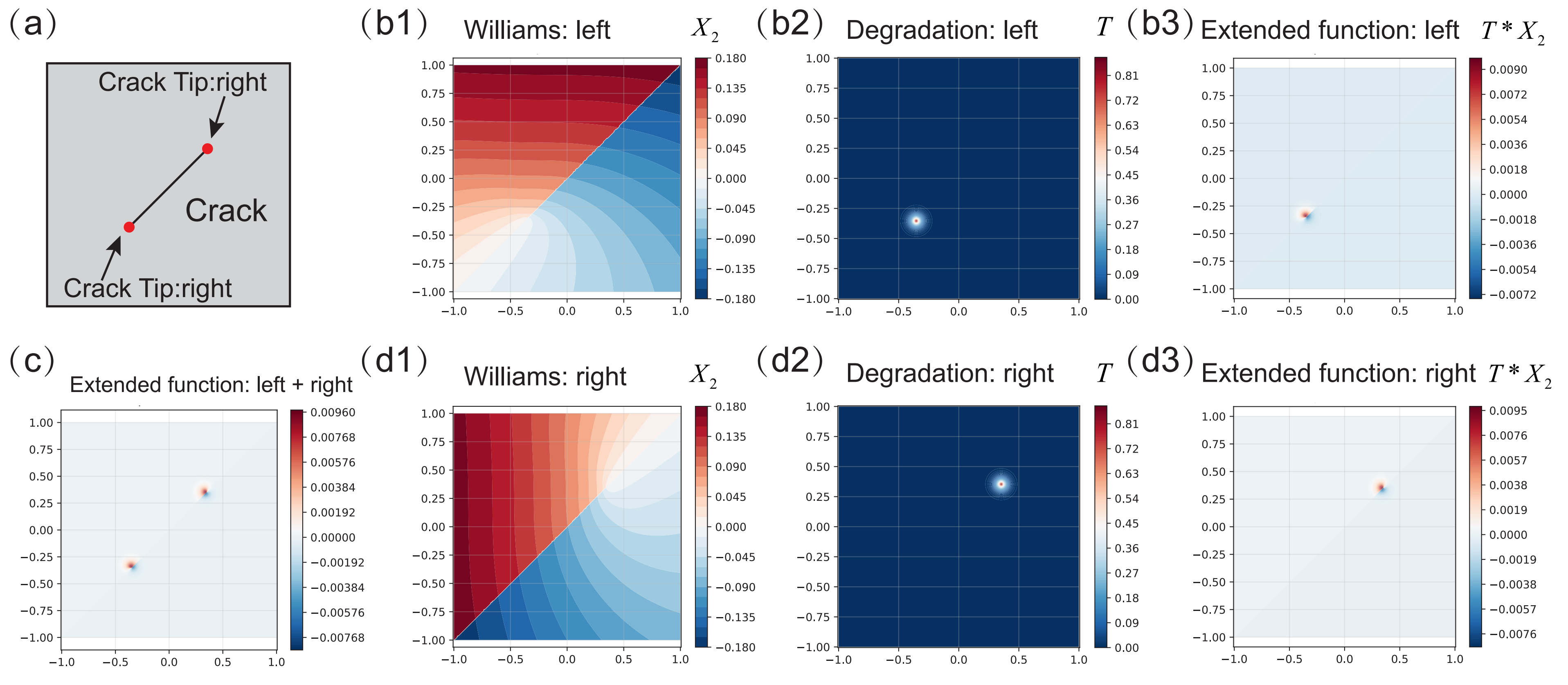

(a)
Crack Tip:right
Crack
Crack Tip:right
(b1) Williams: left X_2
(b2) Degradation: left T
(b3) Extended function: left $T*X_2$
(c) Extended function: left + right
(d1) Williams: right X_2
(d2) Degradation: right T
(d3) Extended function: right $T*X_2$
